# All-Visible-Light-Activated Diarylethene Photoswitches

**DOI:** 10.3390/molecules29215202

**Published:** 2024-11-03

**Authors:** Ruiji Li, Tao Ou, Li Wen, Yehao Yan, Wei Li, Xulong Qin, Shouxin Wang

**Affiliations:** 1 School of Pharmacy, Jining Medical University, Rizhao 276826, China; 15079037989@163.com (L.W.); lwlw1919@163.com (W.L.); xlqin@mail.jnmc.edu.cn (X.Q.); shouxinwang@126.com (S.W.); 2 School of Pharmacy, Binzhou Medical University, Yantai 256603, China; outaoooo@163.com; 3 School of Public Health, Jining Medical University, Jining 272067, China; yanyehao_322@163.com

**Keywords:** photochromism, visible light, diarylethene, design strategy

## Abstract

Photochromic compounds have attracted much attention for their potential applications in photo-actuators, optoelectronic devices and optical recording techniques. This interest is driven by their key photochemical and photophysical properties, which can be reversibly modulated by light irradiation. Among them, diarylethene compounds have garnered extensive investigation due to their excellent thermal stability of both open- and closed-form isomers, robust fatigue resistance, high photocyclization quantum yield and good photochromic performance in both solution and solid phases. However, a notable limitation in expanding the utility of diarylethene compounds is the necessity for ultraviolet light to induce their photochromism. This requirement poses challenges, as ultraviolet light can be detrimental to biological tissues, and its penetration is often restricted in various media. This review provides an overview of design strategies employed in the development of visible-light-responsive diarylethene compounds. These design strategies serve as a guideline for molecular design, with the potential to significantly broaden the applications of all-visible-light-activated diarylethene compounds in the realms of materials science and biomedical science.

## 1. Introduction

**Scheme 1:**
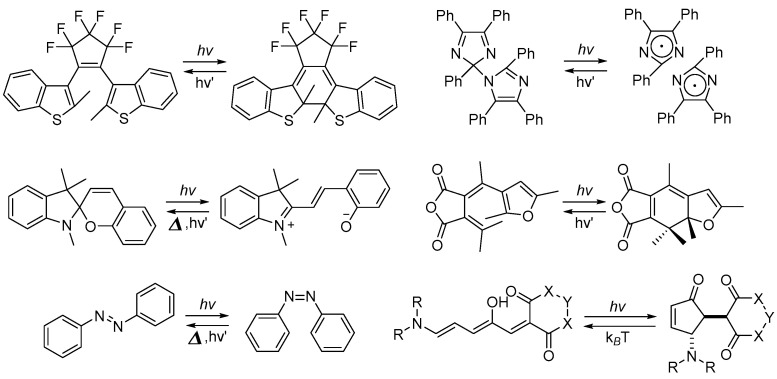
Photochromic reactions of typical photochromic compounds.

Photochromic compounds, characterized by their reversible photoisomerization, color transitions, and modulation of photochemical and photophysical properties upon specific light irradiation, have garnered significant interest for their potential applications in the fields of materials science and biomedical science [1,2,3]. Following extensive research over the years, a multitude of photochromic compounds, including diarylethene (DAE) [4,5], spiropyran [6], azobenzene [7,8], hexaarylbiimidazole [9], fulgide [10], and donor–acceptor Stenhouse adducts (DASAs) [11], have been extensively and intensively investigated (Scheme 1).

The aforementioned photochromic compounds can be classified based on the nature of their photochromic reaction process [12]. For instance, spiropyran, fulgide and diarylethene derivatives exhibit cyclization–cycloreversion reaction processes, azobenzene and stilbene derivatives undergo *trans*-*cis* photoisomerization, hexaarylbiimidazole derivatives demonstrate homolytic cleavage, and salicylideneaniline and dinitrobenzylpyridine (DNBP) participate in proton transfer processes.

Furthermore, photochromic compounds can be classified into T-type and P-type categories depending on the ground-state potential energy barrier between the original stable species and the photogenerated metastable species [13,14]. For T-type photochromic compounds, the photogenerated species are thermally unstable and tend to revert to the initial state in the dark at room temperature. In contrast, P-type photochromic molecules feature thermally stable photogenerated species that are reluctant to return to their initial state in the dark at room temperature.

Additionally, they can be further divided into positive and negative photochromic compounds. Negative photochromic compounds undergo photoisomerization reactions from stable colored isomers to their corresponding metastable colorless isomers [15], whereas positive photochromic compounds perform their photo-induced isomerization reactions from stable colorless isomers to metastable colored isomers.

**Figure 1:**
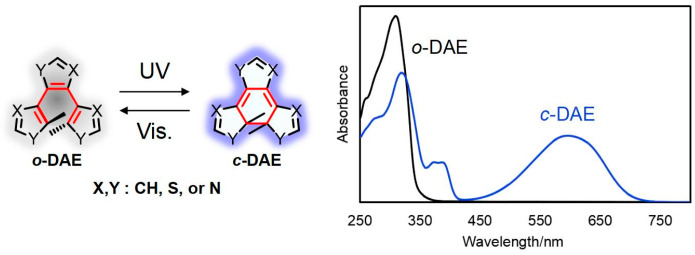
Molecular structures and UV-Vis absorbance spectra of the open- and closed-ring isomers of diarylethenes (*o*-DAE and *c*-DAE).

Diarylethene, a quintessential P-type photochromic compound, has garnered significant research attention owing to its exceptional thermal stability of both open and closed-form isomers, robust fatigue resistance, high photocyclization quantum yield [16], and excellent photochromic performance in both solution and solid phases (Figure 1).

The inaugural research paper on diarylethene compounds was published in 1988 by Irie and Mohri [17]. After more than three decades of investigation, a significant limitation hindering the future application of these compounds is the reliance on UV light for the actuation of the molecular switch in at least one direction. UV light is known to be detrimental to biological tissue and has limited penetration in certain media [4,5]. Consequently, the design and development of diarylethene compounds capable of undergoing photoisomerization upon irradiation with low-energy light are essential for unlocking a wide array of promising applications in biology, medicine, and materials science.

The objective of this review is to provide a summary of recent achievements in the development of photochromic diarylethene compounds that can be triggered in both directions by visible light. Additionally, the design strategies, illustrated with example molecular structures, are also discussed in this review paper.

## 2. Visible-Light-Activated Diarylethenes

### 2.1. Extended π Conjugation

The most effective and straightforward approach to designing visible-light-response diarylethene photochromic materials is to extend the π conjugation of a specific diarylethene compound [18]. The extended π system aims to reduce the HOMO–LUMO gap of the open-form isomer, thereby inducing a red shift in its absorption wavelength. However, as the π conjugation increases, the contribution of the singlet excited state to the central hexatriene unit decreases, potentially suppressing or even eliminating the photoreactivity of the developed diarylethene compounds.

**Scheme 2:**
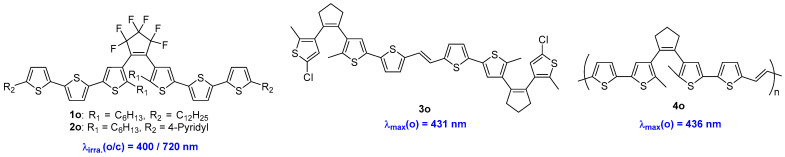
Diarylethene compounds **1o**–**4o** with thiophene side chains.

The first successful attempt to design and synthesize diarylethene compounds, namely **1o** and **2o**, which feature extended π-conjugation and red-shifted absorption without a loss in photoreactivity, was reported by Lehn and coworkers (Scheme 2, Table 1) [19]. The introduction of two additional thiophene units at the 5- and 5′-positions of the dithienylethene led to the red-shift of both maximum absorption wavelengths for both the open- and closed-form isomers. That is, the characteristic absorption wavelengths for the open-form isomers of **1o** and **2o** are 445 nm and 475 nm, respectively. Then, the cyclization reaction of these two compounds can be induced at 400 nm. Even though no relevant quantum yields were reported in this paper, the subsequent publications have verified that the diarylethene compounds with longer π-conjugation at the side chains certainly suppressed their photochromic reactivity and quantum yields [20,21].

Diarylethene compounds **3o** and **4o** (Scheme 2, Table 1) were synthesized via McMurry coupling and ring-opening metathesis polymerization (ROMP), respectively [22]. In this case, the open-form isomers of **3** and **4** exhibited maximum absorption wavelengths of 431 nm (MeCN solution) and 436 nm (CHCl_3_ solution), respectively. Moreover, the thiophene-vinyl component, which is well conjugated with a low energy gap, contributes to the higher photoluminescence quantum yields exhibited by compounds **3o** and **4o**, which are 0.26 and 0.18, respectively. Unfortunately, neither of these two compounds exhibits photochromism upon UV or visible light irradiation. This lack of photochromism could be attributed to the electron density distribution within the thiophene-vinyl potion, rather than the dithienylethene unit. Nonetheless, this design strategy provides a pathway for creating diarylethene compounds with absorption bands in the visible region.

**Scheme 3:**
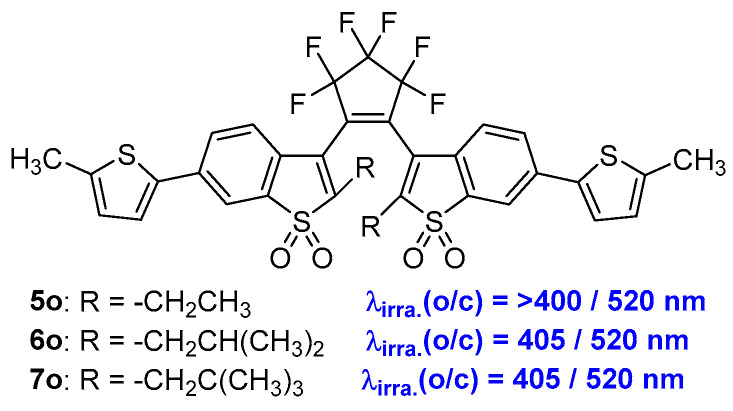
Molecular structures of oxidized bis(benzothienyl)perfluorocyclopentene derivative **5o**–**7o.**

Three diarylethene compounds **5o**–**7o** (Scheme 3, Table 1) by introducing ethyl [23], neopentyl or isobutyl [24] substituents at the reactive carbons (2- and 2′-positions) of an oxidized bis(benzothienyl)perfluorocyclopentene derivative were prepared by Irie and coworkers (Scheme 3). All three of these diarylethene compounds undergo both cyclization and cycloreversion reactions upon irradiation with visible light and exhibit turn-on-mode fluorescent switches. Moreover, compounds **6o** and **7o**, which have neopentyl or isobutyl substituents at the reactive carbons, exhibit cycloreversion quantum yields that are one or two orders of magnitude higher than that of compound **5o**. However, no significant differences in the cyclization and fluorescence quantum yields were observed among these three compounds.

**Scheme 4:**
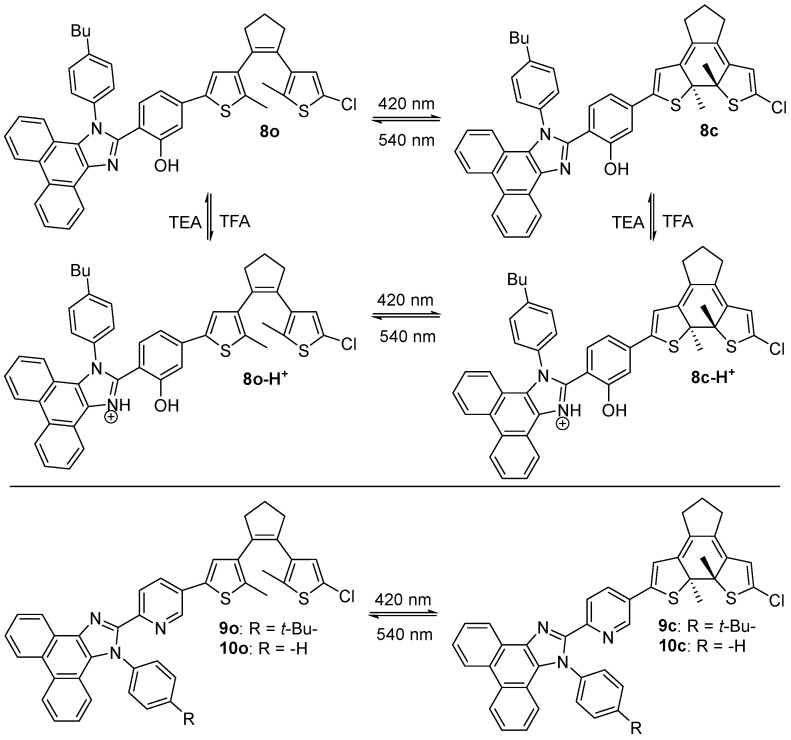
Molecular structures and photochromic reactions of **8o**–**10o** with phenanthroimidazole as the side chain.

All-visible-light-triggered photochromic fluorescent dithienylethene- phenanthroimidazole dyads **8o** [25] and **9o** and **10o** [26] were developed by Wang and coworkers in 2022 (Scheme 4, Table 2). Upon alternate irradiation with 420/540 nm light, compound **8o** exhibited effective photochromic and fluorescent switching in solution, polymethyl methacrylate (PMMA) film, single crystal, and solid powder. Additionally, multi-stimuli responsive chromism and fluorescent switching were achieved with good isomerization reactions when treated with excessive trifluoroacetic acid (TFA). The successfully obtained specific images on filter paper upon the light irradiation of both compound **8o** and acid-treated **8o-H^+^** through a mask demonstrates the development of the photoswitchable dual-mode patterning application.

Upon alternate irradiation with 420 nm and 560 nm light, diarylethene compounds **9o** and **10o** demonstrate efficient absorption and emission spectral changes in solution, PMMA film, solid state, and crystal state and even on filter paper (Scheme 4, Table 2). Additionally, diarylethene compounds **9o** and **10o** exhibit efficient solid-state photochromism and dual-color fluorescence switches their properties, endowing them with potential applications in light-manipulative data storage, anti-counterfeiting and super-resolution imaging. Notably, compound **9o** shows efficient reversible fluorescence ‘‘OFF–ON’’ switching in Hela cells upon all-visible-light irradiation, indicating its potential application in cellular imaging.

**Scheme 5:**
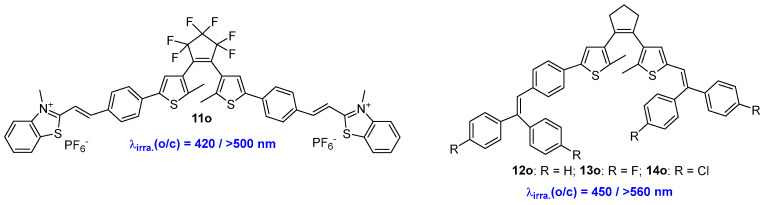
Molecular structures of diarylethene compounds **11o**–**14o.**

A photochromic molecular switch, denoted as **11o** (Scheme 5, Table 3), was developed by linking two photoactive groups, a diarylethene unit and a benzothiazole thiophene styrene, via a π-extended conjugation strategy [27]. With alternate irradiation with light wavelengths at 420 nm and > 500 nm, compound **11o** exhibited excellent photochromic and fluorescent switching in solid-state composite film. This characteristic suggests potential applications in light-recording technology and high-security-level anticounterfeiting systems.

By extending π-conjugation length with aggregated-induced emission units, three all-visible-light triggered fluorescent diarylethene photoswitches (**12o**–**14o**) were designed and prepared by Chi and coworkers (Scheme 5, Table 3) [28]. These three diarylethene compounds (**12o**–**14o**) undergo reversible photochromism in tetrahydrofuran (THF) solution and PMMA films upon alternate irradiation with 450 nm and 560 nm light. Moreover, their extended absorption band in the powdered state enables effective photochromic and fluorescent switching upon similar irradiation, endowing these compounds with versatile switching capabilities in different forms.

Given the visible-light-triggered reversible photochromic and fluorescent switching abilities, diarylethene compounds **12o**–**14o** can be used for all-visible-light-driven patterning applications as an information storage medium on a filter paper. The results indicate that these diaryltehene derivatives have a promising application for visible-light-driven information storage medium, data encryption, and anti-counterfeiting materials.

**Scheme 6:**
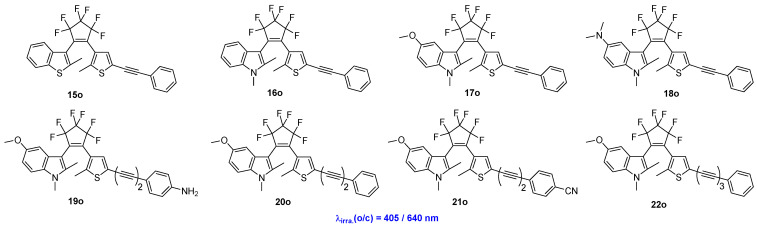
Molecular structures of diarylethene compounds **15o**–**22o** with super-multiplexed Carbow palette.

Hu and coworkers reported a series of photoswitchable diarylethene compounds, designated as **15o**–**22o** (Scheme 6, Table 4), which were synthesized by coupling an asymmetric diarylethene unit with a super multiplexed Carbow [29]. The absorption bands of the open-form isomers of these compounds can extend into the visible region, thereby enabling photocyclization reactions to be triggered by 405 nm light irradiation. Moreover, the cycloreversion reaction can be induced by 640 nm light irradiation. Additionally, live-cell time-lapse imaging of selective organelle dynamics using functionalized Carbow-switches demonstrates the promising applications of these compounds in the biomedical field.

Moreover, with the optimization of both electronic and vibrational spectroscopy, the target diarylethene compounds **15o**–**22o** exhibit excellent visible-light-activated photochromic properties and stimulated Raman scattering (SRS) response, featuring a large frequency shift and signal enhancement. These properties, as described above, endow the compounds with excellent performance in SRS detection applications. This includes reversible and spatially selective multiplexed SRS imaging of various organelles within living cells, characterized by high sensitivity, specificity, biocompatibility, and spatiotemporal selectivity.

**Scheme 7:**
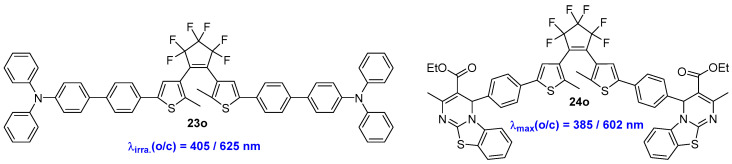
Molecular structures of compounds **23o** and **24o** with different π-conjugation systems as side chains.

An all-visible-light-triggered photoswitch, designated as **23o**, was constructed by Li and coworkers (Scheme 7, Table 5) [30]. Thanks to the electron donating conjugation effect of triphenylamine phenyl (TPAP), the open-form isomer **23o** exhibits a strong response to short-wavelength visible light and undergoes cyclization reaction upon 405 nm light irradiation in both solution and PMMA films. Notably, a near-complete photocyclization reaction, with a ring-closure reaction yield exceeding 96.3%, can be achieved with 405 nm light irradiation. Consequently, this compound can be utilized as optical information media that can be initiated by all visible light, including multilevel data storage, rewritable QR codes, and encryption/anti-counterfeiting applications.

Bisthienylethene-dipyrimido [2,1-*b*][1,3]benzothiazole Triad **24o** was reported by Sun and coworkers (Scheme 7, Table 5) [31]. The open-form isomer of **24o** exhibits an absorption tail that extends to 450 nm in both solution and PMMA film, indicating the potential application for practical solid-state luminescent material by dual-visible-light-induced photoisomerization.

**Scheme 8:**
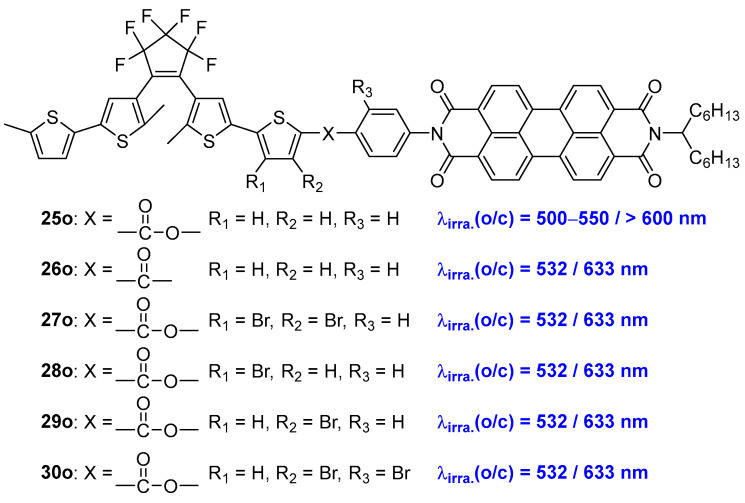
Molecular structures of diarylethene DAE-PBI dyads **25o**–**30o.**

Recently, Fukaminato and coworkers designed and synthesized a series of all-visible-light-activated diarylethene–perylenebisimide dyads by introducing different spacer groups (Scheme 8, Table 5). In particular, the diarylethene derivative **25o**, featuring a covalently attached perylenebisimide (PBI) unit via ester spacer, exhibited reversible cyclization and cycloreversion reactions upon alternate irradiation with green (500−550 nm) and red (>600 nm) light. This characteristic makes it a prime example of an all-visible-light-activatable photoswitch, showcasing its potential for applications in all-visible-light-activated optoelectronic devices and molecular switches [32].

Inferring from the experimental results and theoretical calculations, the mechanism for visible-light-induced cyclization of **25o** involves the generation of the triplet-excited state of the diarylethene unit through multiplicity conversion on the basis of intramolecular energy transfer from the singlet excited state of the PBI unit, and the cyclization subsequently proceeds [33]. However, this compound performed only about 10% photoinduced conversion yield in typical heavy-atom-free solvents and very low photocyclization quantum yield (0.04%) in ethyl acetate solution.

In order to overcome this drawback, diarylethene compound **26o**, which features a ketone group as the spacer between the diarylethene and the PBI units, was prepared [34]. This modification led to reversible photocyclization and photocycloreversion reactions of **26o** upon alternate irradiation with 532 nm and 633 nm laser lights in C_2_H_5_I solvent. Notably, diarylethene compound **26o** achieved nearly complete photoconversion yields in solvents containing heavy atoms and maintained over 50% conversion yield even in heavy-atom-free solvents. Furthermore, the photocyclization quantum yield of compound **26o** (0.4%) was approximately 10 times higher than that of compound **25o** in ethyl acetate solution.

To address the issue of reduced reactivity in solvents containing heavy atoms, diarylethene DAE-PBI dyads (**27o**–**30o**) with heavy bromine atoms at various positions were prepared [35]. The incorporation of bromine atoms [36] significantly enhanced the visible-light-induced cyclization reactivity and increased the photocyclization quantum yields of diarylethene compounds **27o**–**30o** in conventional organic solvents, compared to the previous dyad **25o**, which lacked heavy atoms in the molecule skeleton. The theoretical studies have shown that the involvement of heavy atom atomic orbitals in the LUMOs is crucial for enhancing the reactivity of the visible-light-induced cyclization.

**Table 5:** Photophysical and photochemical parameters of **25o**–**30o** in solution.

Compd.	λ_max_/nm (ε/10^4^ M^−1^ cm^−1^)	*Φ*_oc_	*Φ*_co_	*Φ_f_*	*τ*_f_(ns)	Solvent	*Ref.*
**23o**	354 (10.64)	0.31	0.002			THF	[30]
**24o**	305 (3.91)	0.094	0.006			hexane	[31]
**25o**	354 (10.64)	0.04	1.0 × 10^−3^	0.93	4.2	CCl_4_	[32]
**26o**	-	0.40	0.006	0.97	3.6	CCl_4_	[34]
**27o**	-	0.34	1.5 × 10^−3^	-	-	EtOAc	[36]
**28o**	-	0.35	7.8 × 10^−3^	-	-	EtOAc	[36]
**29o**	-	0.34	1.2 × 10^−3^	-	-	EtOAc	[36]
**30o**	-	0.35	3.9 × 10^−3^	-	-	EtOAc	[36]

λ_max_: absorption maximum; ε: molar absorption coefficient; *Φ*_oc_: cyclization quantum yield; *Φ*_co_: cycloreversion quantum yield; *Φ*_f_: fluorescence quantum yield; *τ*_f_: fluorescence lifetime.

This design strategy provides an innovative and straightforward method for preparing diarylethene switches that can be induced by all-visible light [37,38,39].

**Scheme 9:**
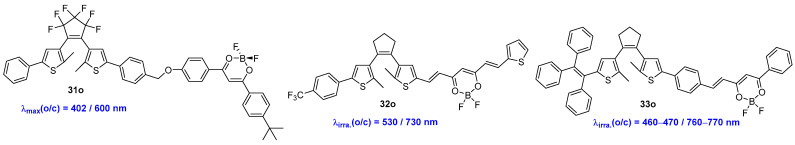
Molecular structures of BF_2_bdk-functionalized dithienylethenes **31o**–**33o.**

Linking a visible light-response difluoroboron β-diketonate to a diarylethene unit is another effective strategy for extending the π-system of diarylethene compound. This modification enables photoswitches that are activated by low-energy light operating in both directions [40]. As shown in Scheme 9 and Table 6, the resulting compound **31o** displays characteristic absorption bands at 402 nm and at around 300 nm, which are attributed to the dibenzoylmethanato boron difluoride complex fluorophore and the diarylethene unit [41]. The absorption band of the corresponding closed form extends into the 500–700 nm range, and the photocycloreversion can be triggered by visible light (>560 nm). Interestingly, the dyad **31o** undergoes efficient photochromic reactions in solution, in nanoparticles (NPs), and in powder solid. Furthermore, the open-form isomer of **31o** exhibits strong blue-colored fluorescence in THF solution with a relatively high fluorescence quantum yield (*Φ*_f_ = 0.37, λ_ex_ = 385 nm), where the pure closed-form isomer is entirely non-fluorescent. This turn-off mode fluorescent property suggests an efficient intramolecular fluorescence resonance energy transfer (FRET) occurring in the closed-form isomer.

Recently, **32o** was prepared by incorporating a difluoroboron β-diketonate (BF_2_bdk) unit and a dithienylethene unit [42]. The resulting **32o** exhibits absorption maxima at 478 nm and 509 nm in toluene, which are derived from the intramolecular charge transfer (ICT) transition [43]. The cyclization and cycloreversion reactions can be effectively initiated upon alternate irradiation with green light (530 nm) and near-infrared (NIR) light (730 nm), respectively. These processes demonstrate significant quantum yields: 0.56 for the cyclization reaction and 0.031 for the cycloreversion reaction. Notably, the fast reversible transformation achieves the photostationary state within only 20 s in PMMA films.

**Table 6:** Photophysical and photochemical parameters of **31o**–**36o** in solution.

Compd.	λ_max_/nm (ε/10^4^ M^−1^ cm^−1^)	Conversion Yield (%)	*Φ*_oc_	*Φ*_co_	*Φ_f_*	Solvent	*Ref.*
**31o**	300, 402	94	-	-	0.37	THF	[41]
**32o**	478 (3.12); 509 (3.68)	-	0.56	0.031	-	toluene	[42]
**33o**	472 (10.2)	-	0.06	0.0023	0.063	toluene	[44]
**34o**	480 (9.37)	-	0.242	0.067	0.52	toluene	[45]
**35o**	454 (8.48)	-	0.161	0.053	0.38	toluene	[45]
**36o**	498 (8.60)	-	0.093	0.058	0.45	toluene	[45]

λ_max_: absorption maximum; ε: molar absorption coefficient; Conversion yield: conversion ratio from open form to closed from at PSS state; *Φ*_oc_: cyclization quantum yield; *Φ*_co_: cycloreversion quantum yield; *Φ*_f_: fluorescence quantum yield.

A compound featuring a triphenylethene and a BF_2_bdk-functionalized dithienylethene, designated as **33o**, has been designed and prepared by Guo and co-workers [44]. Upon alternate irradiation with blue light (460–470 nm) and NIR light (760–770 nm), compound **32o** displays excellent visible light-triggered photochromism and fluorescent switching behavior in both toluene and PMMA films. Interestingly, due to the responsiveness of the BF_2_bdk group to volatile organic amines, compound **32o** displayed distinct fluorescent turn-on and colorimetric sensing performance for volatile *n*-propylamine in THF. It also exhibited fluorescent turn-off and colorimetric sensory behavior towards *n*-propylamine vapor in PMMA films. Consequently, **32o** can be employed as a novel dual sensor for detecting volatile primary or secondary amine vapors in the environment and in biological systems.

**Scheme 10:**
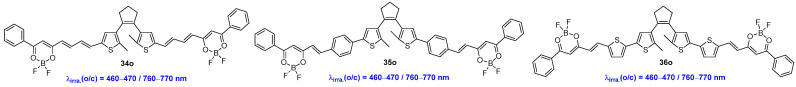
Molecular structures of diarylethene compounds **34o**–**36o** with difluoroboron β-diketonate group.

Dithienylethene-bridged difluoroboron β-diketonate dyes **34o**–**36o** were designed and investigated by Liu, Zhu, Li and co-workers (Scheme 10) [45]. In this study, the difluoroboron β-diketonate group serves a dual role, functioning as an electron acceptor and responding to amine vapors. All of these three compounds display excellent NIR photochromism and fluorescent switching behaviors upon alternate irradiation with blue light (460–470 nm) and NIR light (760–770 nm) in toluene solution and PMMA films. The photochromic properties, including absorption maxima, molar extinction coefficients, optical response rates, and cyclization and cycloreversion quantum yields, can be modulated by the differences in the π-conjugated systems between the difluoroboron β-diketonate moiety and the dithienylethene unit. In addition, these dyes demonstrate an unprecedented triple sensing performance for volatile *n*-propylamine vapor in the PMMA film state.

The 2,2′-positions of the diarylethene compound are involved in bond formation that is photoinduced by cyclization reaction, and indeed, provide a typical method for designing visible-light response switches by extending the π-conjugation. Introducing different units, especially chromophore [46], into the 2 or 2′-position of the diarylethene compound, can induce a hypochromatic-shift of the closed-form isomer compared to the open-form isomer.

**Scheme 11:**
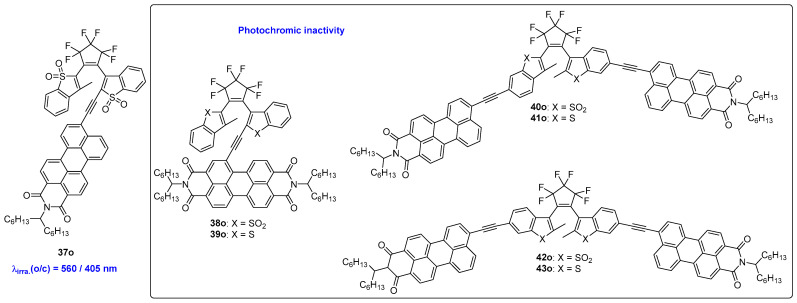
Molecular structures of diarylethene compounds **37o**–**43o** with perylene diimide at different positions.

As shown in Scheme 11 and Table 7, Irie and coworkers successfully designed a visible-light activated diarylethene compound by incorporating a perylene monoimide (PMI) dye at one of the reactive carbons [47]. By alternate irradiation with light wavelengths of 560 nm and 405 nm, compound **37o** exhibited excellent photochromic reactions, switching between its open- and closed-form isomers. The photoconversion efficiencies of compound **37o** are larger than 90% for both cyclization and cycloreversion reactions, but the quantum yields are relatively low at less than 0.01. However, when a perylene diimide (PMI) is placed in the 2-position of the diarylethene unit instead of PDI, the corresponding diarylethene compounds **38o** and **39o** lose their photochromic abilities. Even when two PMI units are introduced to both side chains at the ends of the diarylethene compound, the resulting diarylethene compounds (**40o**–**43o**) also fail to demonstrate photochromism. Density functional theory (DFT) calculations suggest that this phenomenon is due to the localization of the LUMOs on the PMI units, which do not extend to the hexatriene part.

In general, extending the π conjugation to the side chains of the diarylethene compounds can reduce the quantum yields of both cyclization and cycloreversion reactions or even lose photoreactivity. Therefore, another straightforward method for modification of the diarylethene compounds involves constructing the ethene bridge unit [48].

**Scheme 12:**
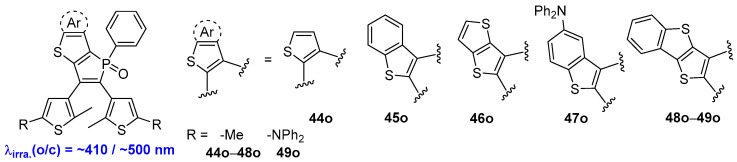
Molecular structures of diarylethene compounds **44o**–**49o** with thieno [3,2-*b*]phosphole oxides units.

In 2017, Yam and coworkers prepared a series of visible-light-induced photochromic thieno [3,2-*b*]phosphole oxides **44o**–**49o** by incorporating a phosphole backbone (Scheme 12) [49]. Upon alternate irradiation with violet (ca. 410 nm) light and green (ca. 500 nm) light, these compounds exhibit excellent thermal irreversibility, robust fatigue resistance, and high photochromic quantum yields (*Φ*_O→C_ = 0.87 and *Φ*_C→O_ = 0.44). The specific properties of this series of diarylethene compounds are likely due to the extended π-conjugated systems integrated into the weakly aromatic phosphole backbone. This integration can reduce the HOMO−LOMO energy gaps of the open-form isomers and extend the excitation wavelength into the visible region. This molecular design strategy offers a practical method for creating diarylethene compounds that are responsive to visible light, boasting excellent photochromic efficiency (Table 8).

**Scheme 13:**
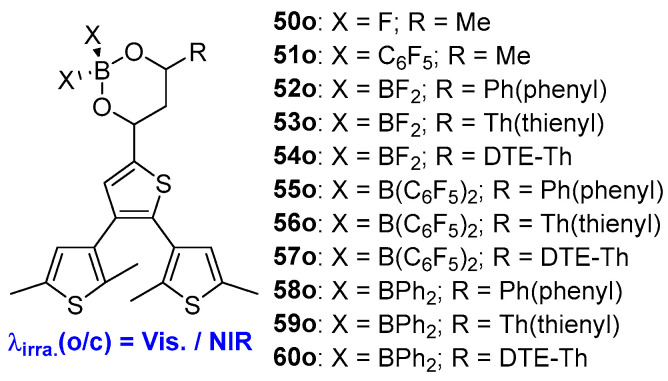
Molecular structures of diarylethene compounds **50o**–**60o** with boron(III) diketonate complexes on the ethene bridge unit.

Due to their diverse photophysical properties and potential applications [50,51,52,53], boron(III) diketonate complexes with a tetra-coordinate boron center have been utilized in conjunction with diarylethene unit. Upon coordination of boron(III) diketonate complexes, Yam and coworkers prepared a series of visible-light-activated diarylethene compounds (**50o**–**60o**, Scheme 13, Table 9). Benefiting from the decrease in LUMO energy localized on the β-diketonatoborane moieties, the photochromic reactions of these compounds can be effectively initiated with visible light irradiation. Interestingly, the closed-form isomers of the di-thienylethene-containing β-diketonateoboranes exhibit a response to near-infrared light [54,55].

**Scheme 14:**
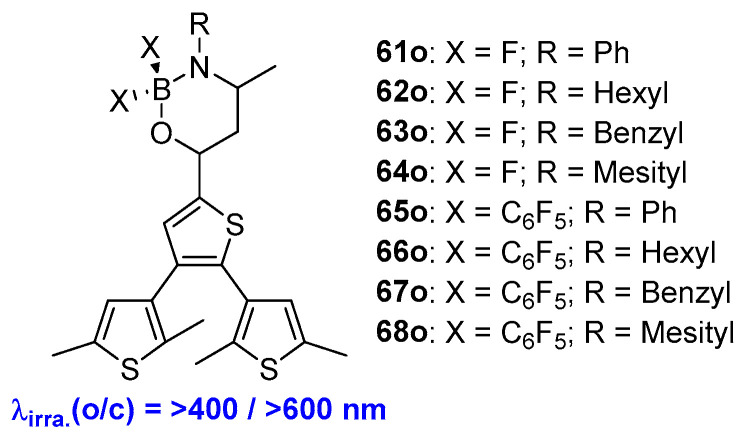
Molecular structures of diarylethene compounds **61o**–**68o** with boron(III)ketoiminates.

By replacing one of the oxygen atoms in β-diketonatoborane with *N*-substituents, the same group developed a series of dithienylethene-containing boron(III)ketoiminates (**61o**–**68o**) [56]. Upon alternate irradiation with visible light at 400 nm for cyclization reactions and 600 nm for cycloreversion reactions, all the compounds exhibit traditional photochromic behaviors (Scheme 14, Table 10). The intramolecular charge transfer (ICT), caused by the electron-rich properties of the *N* atom, may induce a larger bathochromic shift in these compounds. Therefore, introducing an electron donor or an electron acceptor into the diarylethene skeletons to construct a push–pull system is beneficial for inducing a red shift in their corresponding absorption bands.

### 2.2. Push–Pull Systems

Likewise, incorporating an electron donor or an electron acceptor into the diarylethene skeleton to form a push–pull system can lower the HOMO–LUMO gap, resulting in a red shift of its absorption band [57].

**Scheme 15:**
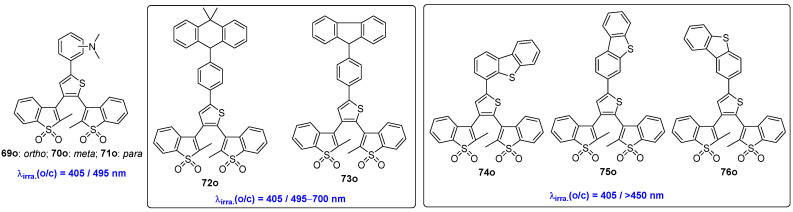
Molecular structures of oxidized bis(benzothienyl)thiophene derivative **69o**–**76o** with different substituent groups on the thiophene bridge unit.

Recently, Liu and coworkers developed three series of 2,3-bis(2-methyl-1-benzothiophene-1,1-dioxide-3-yl)-thiophene derivatives (Scheme 15, Table 11). The three *N*,*N*-dimethylaniline-functionalized diarylethene compounds **69o**, **70o** and **71o** exhibit photochromic reactions and turn-on fluorescence upon visible light irradiation at 405 nm [58]. The corresponding closed-form isomers performed a photoinduced cycloreversion by irradiation with visible light (>495 nm), except for the closed-form isomer (**71c**) of **71o**. When treated with trifluoroacetic acid, the acidic form **71c-H^+^** exhibits a robust photochromic reaction, which can be activated by 405 nm/>495 nm visible light. Moreover, the photochromic reactivity can be quenched again by neutralizing the acid with triethylamine. Notably, this represents the first gated photochromic diarylethene with light-triggered turn-on fluorescence.

Two additional visible-light-triggered fluorescence “turn-on”-mode diarylthene compounds, **72o** and **73o**, were designed with the aid of DFT and time-dependent density functional theory (TD-DFT) theoretical calculation by the same group (Scheme 15) [59]. The introduction of strong electron-donating group, 9,9-dimethyl-9,10-dihydroacridine or Carbazole, shifts the absorption edge of the open-form isomers to the visible light region (~430 nm). Their photochromic reactions can be effectively initiated by alternate irradiation with 405 nm and visible light (495 nm < λ < 700 nm) in toluene solution. Interestingly, the closed-form isomers of both diarylethene compounds exhibit bright yellow intramolecular charge transfer emission with a fluorescence lifetime on the nanosecond scale. Furthermore, both compounds display excellent thermal stability, fatigue resistance, and photostability.

In the same year, Liu and coworkers published another paper detailing three D-A-type, visible-light-induced fluorescence “turn-on”-mode diarylethene compounds **74o**–**76o**. These compounds were synthesized by integrating a dibenzo[*b*,*d*]thiophene donor into a 2,3-bis(2-methyl-1-benzothiophene-1,1-dioxide-3-yl)-thiophene acceptor (Scheme 15, Table 11) [60]. Similar to the aforementioned molecules, the introduction of the dibenzo[*b*,*d*]thiophene donor helps to extend the molecular π conjugation and increase intramolecular charge transfer, which is beneficial for red-shifting the absorption of the open-form isomers to the visible light region. These compounds **74o**–**76o** were cyclized into closed-form isomers with relatively high photoluminescence quantum yields (0.18–0.21) upon 405 nm visible light irradiation. The initial open-form isomers were then obtained with visible light (>450 nm) irradiation of the corresponding closed-form isomers. Additionally, these three molecules also exhibit good thermal stability, fatigue resistance and photostability.

Typically, the all-visible-light triggered “turn-on”-mode fluorescent photo switches described above can be utilized as gated photochromic materials and molecular probes for super-resolution fluorescence imaging in biological cells.

**Scheme 16:**
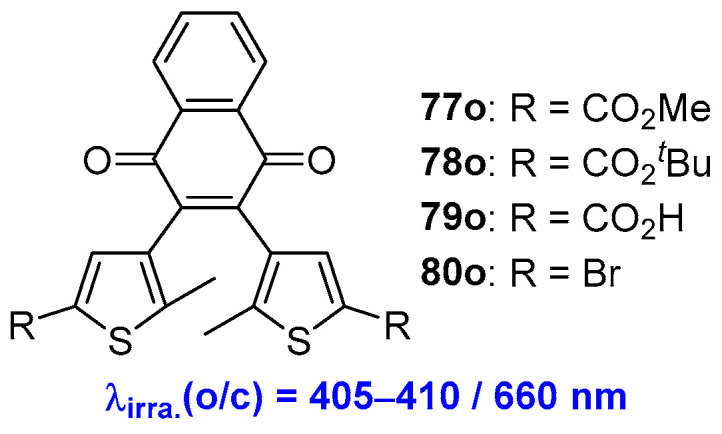
Molecular structures of naphthoquinone-based diarylethene compounds **77o**–**80o.**

Photochromic diarylethene compounds containing quinone functionalities were developed by Patel and coworkers (Scheme 16) [61,62]. The naphthoquinone-based diarylethene photochromes **77o**–**80o** undergo photocyclization reactions from open-form isomers to closed-form isomers upon visible light irradiation at 405–410 nm. However, upon irradiation with 660 nm visible light, the presence of absorption at about 440 nm indicates that both open-form and closed-form isomers coexist in the solution. The relatively low photoswitching efficiency of these diarylethene compounds is explained by the overlap of absorption bands between the open-form and closed-form isomers, leading to incomplete conversion from open-form and closed-form isomers.

**Scheme 17:**
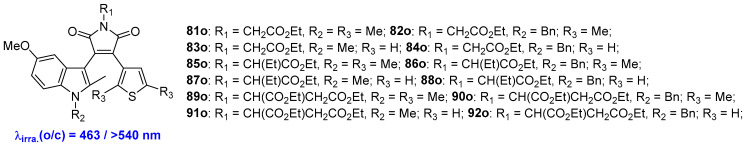
Molecular structures of diarylethene-amino acid photochromic fluorescent hybrids **81o**–**92o.**

A series of diarylethene-amino acid photochromic fluorescent hybrids, **81o**–**92o**, were developed by Dubonosov and coworkers (Scheme 17, Table 12) [63]. Irradiation of the hexane solutions of compounds **81o**–**92o** with 463 nm light induces a rearrangement into the colored closed-form isomers, which absorb at longer wavelengths in the spectrum, ranging from 552 nm to 574 nm. Subsequently, rapid cycloreversion reactions can be triggered by irradiation with visible light (>540 nm), accompanied by a color disappearance process.

**Scheme 18:**
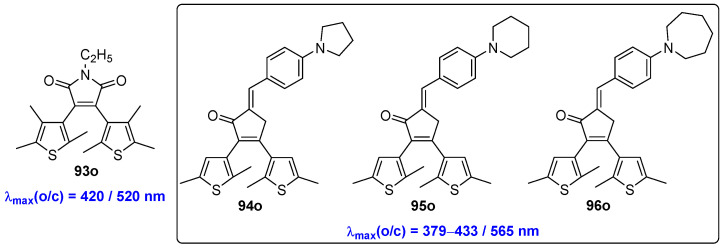
Molecular structures of diarylethene compounds **93o**–**96o** with different antennas on ethene bridge unit.

The alkylated diarylethene molecule **93o** was designed by Miyaura and coworkers (Scheme 18, Table 13) [64]. This diarylethene compound **93o** exhibits a maximum absorption wavelength at approximately 410 nm, with the absorption edge of the open-form isomer reaching 500 nm. Consequently, both the cyclization and cycloreversion reactions of the diarylethene compound **93o** can be initiated by visible light. The introduction of a push–pull system between carbonyl and dimethylamino groups in the ethene bridge of diarylethenes **94o**–**96o** results in intense absorption maxima from 379 nm to 433 nm, which very much depend on the solvent and the ring size of azoheterocyclic moiety (Scheme 18) [65]. This characteristic enables the photoinduced cyclization to occur upon exposure to visible light in non-polar solvents. In contrast, in polar solvents, the major photoreaction is *E*/*Z*-isomerization of the arylidene fragment. Therefore, diarylethene compounds **94o**–**96o**, which feature a π-conjugated push–pull system, exhibit solvent-dependent photochromic properties.

**Scheme 19:**
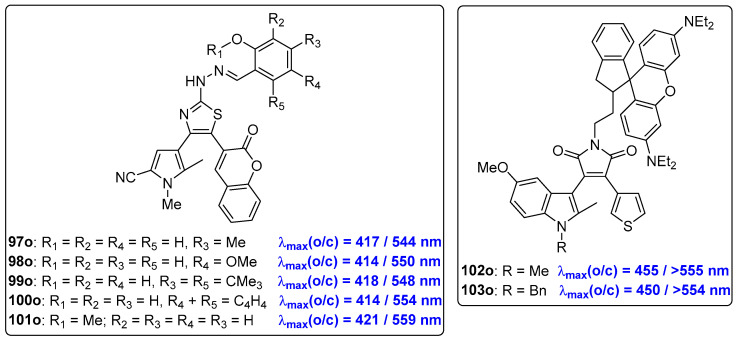
Molecular structures of bifunctional diarylethene compounds **97o**–**103o.**

Coumarinyl(pyrrolyl)ethenes with a 1,3-thiazole bridge, **97o**–**101o**, undergo photocyclization reactions when exposed to light at 365 nm in acetonitrile, and their photocycloreversion reactions can be induced by irradiation with visible light at 540 nm (Scheme 19, Table 14) [66]. The maximum absorption wavelength at approximately 420 nm offers the potential for visible-light-induced photocyclization reaction. Moreover, compounds **97o**–**99o** can serve as multifunctional sensors for detecting Pd^2+^, CN^−^ and F^−^ ions with selective chromogenic activity. This property endows compounds **97o**–**99o** with the potential to be used as logic circuits, responding to different input signals and producing an output signal. Additionally, bifunctional compounds **102o** and **103o**, which contain a photochromic indolyl(thienyl) diaryltehene with a pyrroldione bridge directly linked by a dimethyiene spacer to an ionochromic rhodamine moiety, were developed by the same research group (Scheme 19) [67]. The absorption bands of both compounds **102o** and **103o**, located at 450–455 nm in toluene solution, provide the potential for visible-light-induced photochromic reactions.

### 2.3. Energy Transfer

In addition to the design strategy of diarylethene compounds with extended π-conjugation and a push–pull system to directly address the absorption band into the visible region, intramolecular or intermolecular energy transfer from a sensitizer to the photoswitch is often employed to develop visible-light-triggered diarylethene compounds. In this case, the sensitizer can absorb radiation in the visible or even NIR region and transfer the higher excitation energy to the diarylethene photoswitch, thereby initiating the photochromic reaction [68]. The sensitizer can be a triplet sensitizer, a multi-photon absorber/emitter, or an upconverting nanoparticle.

**Figure 2:**
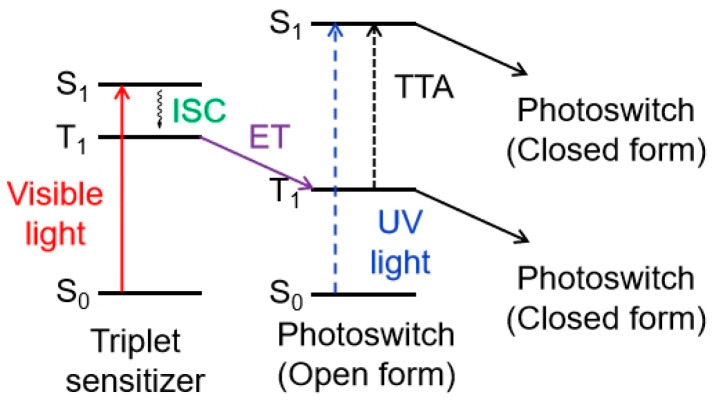
Energy transfer from a triplet sensitizer to the photoswitch.

Using triplet sensitization to achieve all-visible-light photochromism is also an efficient design strategy. For easy energy transfer, the S_1_–S_0_ energy gap of the sensitizer must be lower than that of the diarylethene photoswitch to utilize low-energy excitation light. Additionally, the energy of the triplet state of the sensitizer should be larger than the T_1_ energy level of the diarylethene photoswitch (Figure 2).

The incorporation of transition metal ions into the diarylethene skeleton facilitates visible-light-triggered switching through intramolecular metal-to-ligand charge-transfer (MLCT) processes [69,70,71,72,73]. However, due to the high cost of the metal ligands, concerns about metal contamination [74], low cycloreversion quantum yields of MLCT-based diarylethene compounds [75], and poor triplet-sensitization efficiency [76,77], relatively few visible-light-triggered diarylethene photoswitches have been successfully developed using this design method.

**Scheme 20:**
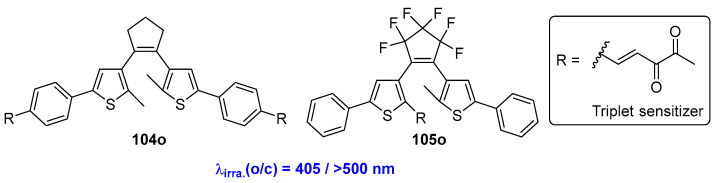
Molecular structures of diarylethene compounds **104o** and **105o** with a triplet sensitizer.

In order to overcome the drawbacks described above, a diarylethene photoswitch covalently linked to two small biacetyl triplet sensitizers was reported by Hecht and coworkers (Scheme 20, Table 15) [78]. Benefiting from the elongated conjugation and effective intramolecular triplet energy transfer from the biacetyl termini to the diarylethene core, the open-form isomer **104o** performs with a strong maximum absorption wavelength at 390 nm. The photoinduced cyclization reaction can be initiated by visible-light (λ_irra_. = 405 nm) irradiation. The ring-closed isomer **104c** can readily be converted back to its open form **104o** by irradiation with long-wavelength light (λ_irra_. > 500 nm). The triplet sensitization process was experimentally demonstrated by examining the oxygen sensitivity of the photochromic reaction. Moreover, a diarylethene compound bearing one biacetyl at the photoreactive carbon was also developed by the same group [79]. Although the photocyclization reaction of diarylethene compound **105o** from the open- to closed-form isomer can be induced by 405 nm, the maximum absorption wavelength of the open-form isomer of **105o** was located at 289 nm, with a shoulder absorption at 265 nm. This further confirms the dominant role of the triplet sensitization in the photochromism.

**Scheme 21:**
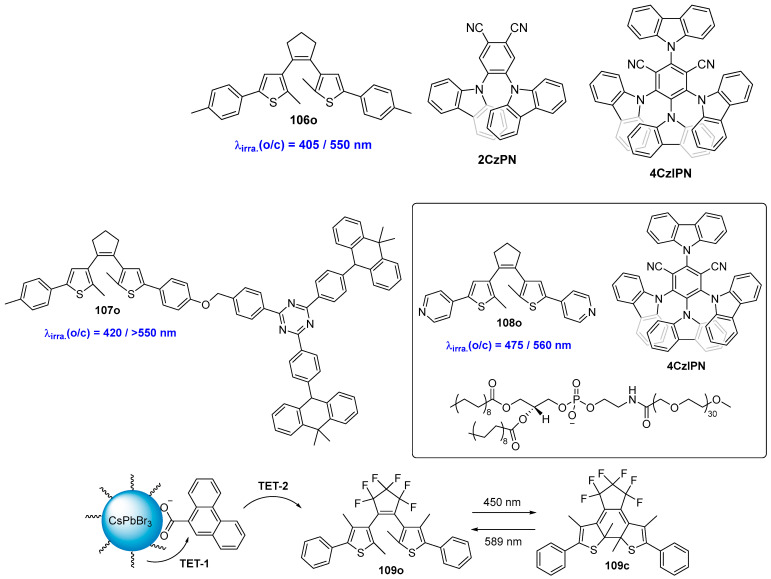
Molecular structures of diarylethene compounds **106o**–**109o** with TTET or TET process.

The all-visible-light-induced photochromism of diarylethene compounds featuring a narrow singlet-triplet energy gap (∆*E*_ST_) materials as a sensitizer was reported by Tian, Zhang and coworkers (Scheme 21) [80]. Benefiting from the narrow ∆*E*_ST_ that matches the triplet–triplet energy transfer (TTET) process, the all-organic visible-light photochromism of diarylethenes has been successfully achieved with high efficiency and photofatigue resistance. Moreover, the wavelength of the illuminating light can be easily modulated by changing the related sensitizers. The all-visible-light “write-and erase” photochromic system can also be fabricated by using **106o**/2CzPN and **106o**/4CzIPN in PMMA films. One year later, the same research group developed an all-visible-light diarylethene system, referred to as **107o**, by introducing a building-block design strategy with a narrow ∆*E*_ST_ sensitizer into the diarylethene core [81]. The photochromic performance of diarylethene **107o** can be initiated through alternate irradiation with 420 nm/>550 nm light, demonstrating high efficiency and robust fatigue resistance. Furthermore, this compound is also suitable for all-visible-light patterning applications, making it viable candidate for use in information storage media. An amplifying dual-visible-light diarylethene system made by combining diarylethene compound **108o**, a triplet sensitizer (4CzIPN), and an amphiphile (mPEG-DSPE) was developed by Zhang and coworkers [82]. This combination resulted in a visible-light switchable micelle, which features distance dependence and environmental sensitivity in the Dexter-type triplet-triplet process. As a result, an approximately 10-fold enhancement of the TTET efficiency for photochromism was achieved. Additionally, an amplified fluorescence on/off contrast upon bidirectional visible-light excitation at 470/560 nm was detected in full water media. Furthermore, fluorescence confocal imaging of Hela cells incubated with this diarylethene system can be realized through alternate irradiation with 475/560 nm light.

Recently, Wu and coworkers reported an all-visible-light-activated molecular isomerization reaction, sensitized by lead halide perovskite nanocrystals (CsPbBr_3_ NC-PTA) [83]. This reaction capitalizes on the triplet energy transfer (TET) from CsPbBr_3_ NC-PTA to the organic photoswitch, enabling the diarylethene molecule **109o** to exhibit photoisomerization reaction under 5 mW illumination with a 450 nm laser. Subsequently, the colored closed-form isomer can be readily reverted to the colorless open-form by irradiation with a 589 nm laser. This typical photochromic reaction facilitates applications such as light-induced information coding and patterning. The same methodology was also applied to control the photoluminescence emission from the nanocrystals through light stimulation [84].

An alternative method for obtaining a visible-light-triggered molecular switch involves populating the singlet excited state (S_1_) through a triplet–triplet annihilation (TTA) mechanism [85,86,87]. In this process, two molecular switches, both in the T_1_ state, interact such that one is promoted to the S_1_ state while the other returns to the S_0_ state. To date, several examples have been reported utilizing perylene [88,89] or metal complex [90,91] triplet acceptors to achieve visible-light-triggered photochromism.

**Scheme 22:**
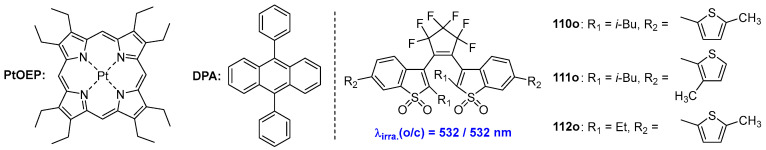
Molecular structures of diarylethene compounds **110o**–**112o** with TTA mechanism.

Recently, a series of diarylethene compounds (**110o**–**112o**) featuring visible-light-induced isomerization were developed utilizing the well-studied upconversion pair 9,10-diphenylanthracence (DPA) and platinum octaethylporphyrin (PtOEP) [92]. Both the ring-opening and ring-closing reactions of these compounds can be triggered by a single green light source at 532 nm (Scheme 22).

### 2.4. Negative Photochromism

In most cases, the photochromic compounds described above undergo photochromic reactions from stable colorless isomers to metastable colored isomers upon light irradiation. These compounds refer to the positive type photochromic compounds. Furthermore, the photochromic molecules perform their isomerization reactions from stable colored isomers to their corresponding metastable colorless isomers by light excitation and can be recognized as negative photochromic molecules [5,93].

The most typical characteristic for negative photochromic compounds is the transformation of the stable colored isomer into the metastable colorless or lightly colored isomer upon exposure to visible or near-infrared light irradiation. Subsequently, the reversible reaction can be triggered by short-wavelength light irradiation or through a thermal back reaction in the dark [5,93]. Therefore, another ingenious method for designing visible-light- or even NIR-light-responsive diarylethene compounds is to impart them the capability of negative photochromism.

**Scheme 23:**
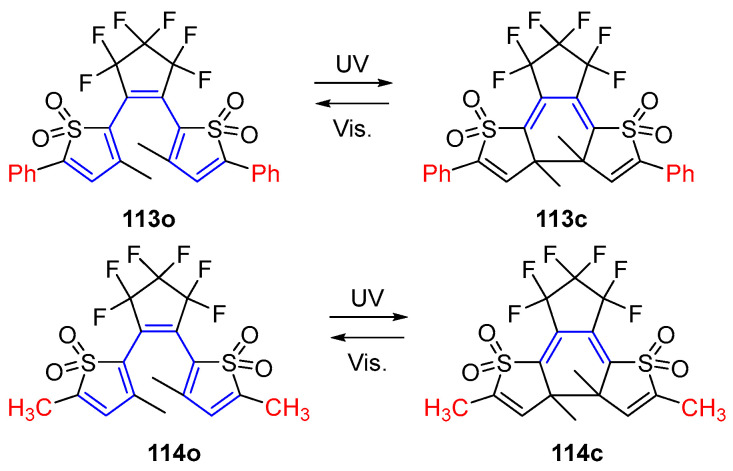
Molecular structures and photochromic reactions of **113o** and **114o.**

The first negative photochromic compound based on a diarykethene skeleton was reported by Irie and coworkers in 2008 [94]. As shown in Scheme 23 and Table 16, the molecular design strategy is to connect the oxidized thiophene rings to the central ethene moiety through the 2-position. This typical molecular design method causes the open-form isomer to have relatively longer π-conjugation length than the photoinduced closed ring isomer. This specific molecular π-conjugation length changes lead to the negative photochromism or “invisible photochromism” of diarylethene compounds upon light irradiation.

As the open form with a relatively longer π-conjugation length, the absorption maximum of **113o** is located at 356 nm with a slight yellow 1,4-dioxane solution. Then, a new absorption band appeared in the UV region, along with a decoloration process upon visible light (>430 nm) irradiation. Furthermore, the irradiation of 1,4-dioxane solution of **113c** with UV light (λ = 313 nm) triggers the colored reaction from colorless to pale yellow along with the recovery of the absorption band at 356 nm which corresponds to the open form isomer **113o**. The formation of the closed-form isomer, along with the negative photochromic reaction, was further confirmed by ^13^C NMR spectroscopy and X-ray crystallographic analysis.

By changing the substituents from phenyl units to methyl groups at the 5-position of both oxidized thiophene units, diarylethene compound **114o** in 1,4-dioxane is colorless with the absorption band at the UV region. Similar to **113o**, the light (λ = 390 nm) induced photoisomerization causes a decrease in absorption at 317 nm and the appearance of a new absorption band at 284 nm. Then, the initial absorption band was recovered with UV light (λ = 284 nm) irradiation. Interestingly, there is no obvious color change with the photoisomerization reaction of diarylethene compound **114** in the 1,4-dioxane solution. This is another phenomenon that is defined as “invisible photochromism”. The unconventional photochromic behaviors of compounds **113** and **114** have also been elucidated with the help of the theoretical investigation by DFT calculation [95].

**Scheme 24:**
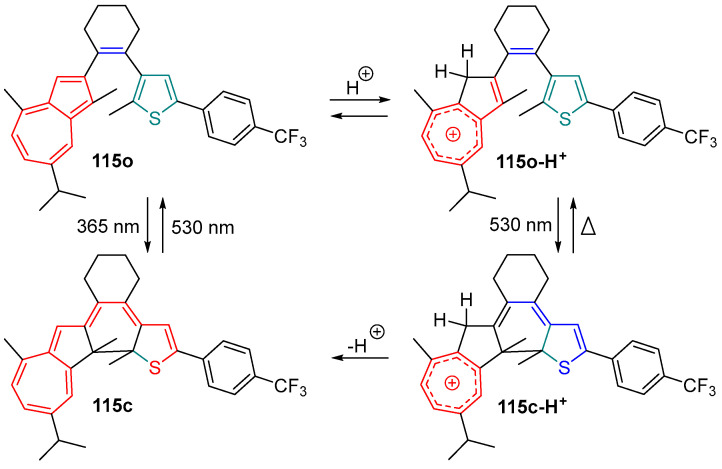
Proton-gated photochromism of azulene-based diarylethene compound **115** in its open (**top**) and closed (**bottom**) isomers and their conjugated acids.

Another negative compound based on the diarylethene skeleton, called the photo-gated [96,97] compound, was designed and reported by Hecht and coworkers [98]. In the diarylethene compound **115**, an acid-sensitive azulene moiety as one of its side aryl groups was introduced to the molecular skeleton (Scheme 24). Upon light irradiation (λ = 365 nm), diarylethene compound **115** exhibits a positive photochromic reaction with the color change from a colorless open form (**115o**) to a colored closed form (**115c**). However, the absorption band of DAE **115o** was changed significantly with the addition of TFA. In this case, the absorption band of the acid-treated **115o** decreased at around 300 nm, and a new strong absorption band extending from 350 to 550 nm appeared. This typical optical property indicates that the protonated **115o-H^+^** has the potential for displaying negative photochromic behavior. With the irradiation of visible light at the wavelength of 546 nm, the decoloration process of **115o-H^+^** cyclohexane solution was observed, the absorption band at visible region decreased, and another absorption band at ultraviolet region became greatly prominent. This phenomenon indicates the negative photochromism of the protonation compound **115o-H^+^**. Subsequently, the closed isomer **115c-H^+^** undergoes a thermal ring-opening reaction with a half-life of around 7 min at room temperature. This research not only opens a new avenue for designing diarylethene compounds with visible light excitation but also provides an artful designing method for enhancing the photochemical efficiency compared to the original untreated molecule as well as creating multiple-stimuli responsive molecular switches [99].

A C-shaped hydrocarbon cethrene composed of seven fused benzenoid rings, which can be recognized as a diarylethene derivative, shows an electrocyclization reaction via both photochemical and thermal processes. However, the facile oxidation reaction forced the closed-ring form to be a planar hydrocarbon, and the intermediate compound could not be isolated or detected. The oxidation reaction quenched the photochromic reversible transformation from closed-ring form to initial open-ring form [100,101,102,103]. Nevertheless, two methyl substituents installed in the fjord region instead of two hydrogen atoms improved not only the stability of the system against oxidation reaction but also the expeditiousness of the synthesis. This design strategy enabled compound **116** to show reversible transformation between the open- and closed-form isomers by light irradiation.

**Scheme 25:**
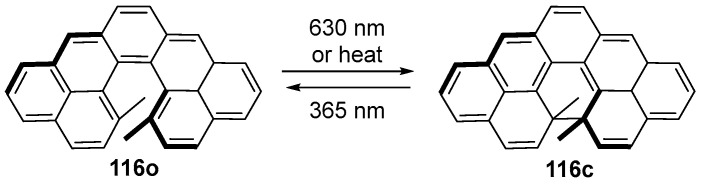
Molecular structures and photochromic reactions of C-shaped hydrocarbon cethrene-type diarylethene **116o.**

Based on the Woodward–Hoffmann rules, the conrotatory electrocyclic ring-closure and ring-opening reaction of compound **116** can be efficiently mediated by visible (λ = 630 nm) and ultraviolet (λ = 365 nm) light, respectively (Scheme 25). Along with the photochemical reaction, the blue solution of the metastable open form (**116o**) was transformed to a colorless solution of the stable closed form upon irradiation at 630 nm. The reversible photoreaction from colorless closed form to colored open form can be induced with 365 nm light irradiation [104]. Strictly speaking, compound **116o** could not be recognized as a negative photochromic compound because the colored open form was the metastable isomer.

### 2.5. Others

Over the years, in the pursuit of developing all-visible-light-triggered diarylethene compounds, various design strategies have emerged. These include the use of upconverting nanoparticles [105,106], multiphoton absorption [107,108], supermolecular self-assembly [109,110,111], coordinative cages [112], intramolecular proton transfer [113,114], and so on.

Given the scarcity of reports on visible-light-triggered photochromic diarylethene compounds based on the aforementioned design strategies over the past five years, and considering that research prior to 2020 has already been summarized in two mini-review papers [115,116], we will not further discuss these design strategies in this context.

## 3. Conclusions and Perspectives

Due to the specific properties, such as excellent thermal stability of both open- and closed-form isomers, robust fatigue resistance, high photocyclization quantum yields, and reliable photochromic performance in both solution and solid phases, diarylethene compounds have been extensively investigated for applications in materials science and biomedical sciences. However, the fact that the photochromic reaction from open-form isomers to closed-form isomers is typically induced by ultraviolet light restricts their broader applications. Therefore, the development of all-visible-light-activated diarylethene compounds is essential for opticelectronic devices and photocontrolled biological applications. This is because visible light excitation offers advantages such as low phototoxicity, high permeability, and a low background signal.

In this review, we have examined several effective design strategies, such as extended π-conjugation systems, push–pull systems, energy transfer, and negative photochromism, have been utilized to develop diarylethene compounds responsive to visible light in both directions. However, several challenges and limitations remain to be addressed: (1) Designing all-visible-light-activated diarylethene compounds with high stability and photoisomerization efficiency with respect to both LED and sunlight is essential for enriching the design strategies and expanding their applications in portable medical devices. (2) Improving the photocyclization and photocycloreversion quantum yields in both solution and solid/crystal states is a second essential property to be enhanced. (3) Adjusting the absorption bands of both open-form isomers and closed-form isomers to long-wavelength light, such as red or even NIR light, is crucial for applications requiring light penetration into (bio)materials without causing radiation damage. (4) Cytotoxicity is another critical factor, especially for applications in photopharmacology. (5) Multi-responsibility, such as responsiveness to pH, singlet oxygen, and human immune cell markers, is also pivotal for their utility in biomedical field. (6) Integrating nonlinear optical features into diarylethene compounds that can be activated with low-energy light presents another challenge. (7) Expending the applications of all-visible-light-activated diarylethene compounds into microelectronic devices and biomedical systems is a key goal for our future research.

The development of new types of all-visible-light-activated diarylethene compounds, achieved through ingenious molecular design strategies and structural modifications to address the aforementioned challenges, is poised to have a profound impact on the fields of materials science and biomedical science. Undoubtedly, the development of these innovative molecules will spark a significant scientific and technological revolution, paving the way for transformative advancements across various fields.

## Figures and Tables

**Table 1:** Photophysical and photochemical parameters of **1o**–**7o** in solution.

Compd.	λ_max_/nm (ε/10^4^ M^−1^ cm^−1^)	*Φ*_oc_	λ_PL_	*Φ*_f_	Solvent	*Ref.*
**1o**	372 (5.3), 445	-	-	-	cyclohexane	[19]
**2o**	391(5.6), 475	-	-	-	cyclohexane	[19]
**3o**	431 (-)	-	490	0.26	MeCN	[22]
**4o**	436 (-)	-	499	0.18	CHCl_3_	[23]
**5o**	330 (2.4), 374 (2.5)	0.23	-	-	1,4-dioxane	[24]
**6o**	330 (2.3), 375 (2.3)	0.30	-	-	1,4-dioxane	[24]
**7o**	330 (2.3), 377 (2.6)	0.32	-	-	1,4-dioxane	[24]

λ_max_: absorption maximum; ε: molar absorption coefficient; *Φ*_oc_: cyclization quantum yield; λ_PL_: fluorescence maximum; *Φ*_f_: fluorescence quantum yield.

**Table 2:** Photophysical and photochemical parameters of **8o**–**10o** in solution.

Compd.	λ_max_/nm (ε/10^4^ M^−1^ cm^−1^)	*Φ*_oc_	*Φ*_co_	λ_PL_	*Φ*_f_	Solvent	*Ref.*
**8o**	372 (6.33)	0.34		466	0.1941	CHCl_3_	[25]
**8c**	516 (2.57)		0.02	-	-	CHCl_3_	[25]
**8o+H^+^**	378	-		476	0.0644	CHCl_3_	[25]
**8c+H^+^**	749 (0.68); 754 (0.67); 751 (0.22)	-		-	-	CHCl_3_	[25]
**9o**	375 (5.62)	0.30		433	0.0973	THF	[26]
**9c**	518 (2.56)		0.01	-	-	THF	[26]
**10o**	374 (4.68)	0.40		436	0.0767	THF	[26]
**10c**	517 (1.86)		0.02	-	-	THF	[26]

λ_max_: absorption maximum; ε: molar absorption coefficient; *Φ*_oc_: cyclization quantum yield; *Φ*_co_: cycloreversion quantum yield; λ_PL_: fluorescence maximum; *Φ*_f_: fluorescence quantum yield.

**Table 3:** Photophysical and photochemical parameters of **11o**–**14o** in solution.

Compd.	λ_max_/nm (ε/10^4^ M^−1^ cm^−1^)	*Φ*_oc_	*Φ*_co_	λ_PL_	*Φ*_f_	Solvent	*Ref.*
**11o**	259, 429	0.06	0.002	554	0.02	MeCN	[27]
**12o**	354 (5.34)	0.43	0.04	-	0.0072	THF	[28]
**13o**	355 (7.48)	0.39	0.07	-	0.0088	THF	[28]
**14o**	360 (8.64)	0.41	0.10	-	0.0409	THF	[28]

λ_max_: absorption maximum; ε: molar absorption coefficient; *Φ*_oc_: cyclization quantum yield; *Φ*_co_: cycloreversion quantum yield; λ_PL_: fluorescence maximum; *Φ*_f_: fluorescence quantum yield.

**Table 4:** Photophysical and photochemical parameters of **15o**–**22o** in CHCl_3_ solution.

Compd.	λ_max_/nm (ε/10^4^ M^−1^ cm^−1^)	Solvent for Absorption	Conversion Yield (%)	Solvent for Conversion Yield	*Ref.*
**15o**	309 (3.00)	DMSO	38	CHCl_3_	[29]
**16o**	312 (2.75)	DMSO	72	CHCl_3_	[29]
**17o**	307 (2.79)	DMSO	83	CHCl_3_	[29]
**18o**	313 (2.66)	DMSO	-	CHCl_3_	[29]
**19o**	358 (4.98)	DMSO	86	CHCl_3_	[29]
**20o**	337 (3.30)	DMSO	85	CHCl_3_	[29]
**21o**	350 (3.43)	DMSO	92	CHCl_3_	[29]
**22o**	359 (3.83)	DMSO	93	CHCl_3_	[29]

λ_max_: absorption maximum; ε: molar absorption coefficient; conversion yield: conversion ratio from open form to closed from at PSS state.

**Table 7:** Photophysical and photochemical parameters of **37o and 38o** in solution.

Compd.	λ_max_/nm (ε/10^4^ M^−1^ cm^−1^)	Conversion Yield (%)	*Φ*_oc_	*Φ_f_*	solvent	*Ref.*
**37o**	543 (4.39)	97	6.4 × 10^−4^	0.016	1.4-dioxane	[43]
**38o**	554 (4.64)	>99	3.1 × 10^−4^	0.11	1.4-dioxane	[43]

λ_max_: absorption maximum; ε: molar absorption coefficient; Conversion yield: conversion ratio from open form to closed from at PSS state; *Φ*_oc_: cyclization quantum yield; *Φ*_f_: fluorescence quantum yield.

**Table 8:** Photophysical and photochemical parameters of **44o**–**49o** in benzene solution.

Compd.	λ_max_/nm (ε/10^4^ M^−1^ cm^−1^)	λ_em_/nm	*Φ*_oc_	*Φ*_co_	Conversion Yield (%)	*Φ_f_*	Solvent	*Ref.*
**44o**	385 (0.59)	523	0.50	0.10	88	0.064	benzene	[45]
**45o**	333 (0.81), 410 (0.699)	543	0.87	0.44	51	0.032	benzene	[45]
**46o**	327 (0.792), 414 (0.975)	542	0.77	0.43	85	0.048	benzene	[45]
**47o**	310 (2.75), 419 (0.954)	562	0.35	0.042	78	0.011	benzene	[45]
**48o**	342 (0.656), 421 (1.64)	552	0.23	0.084	83	0.056	benzene	[45]
**49o**	310 (2.17), 411 (3.61)	645	0.35	0.087	84	0.006	benzene	[45]

λ_max_: absorption maximum; ε: molar absorption coefficient; *Φ*_oc_: cyclization quantum yield; *Φ*_co_: cycloreversion quantum yield; conversion yield: conversion ratio from open form to closed from at PSS state; *Φ*_f_: fluorescence quantum yield.

**Table 9:** Photophysical and photochemical parameters of **50o**–**60o** in benzene solution.

Compd.	λ_max_/nm (ε/10^4^ M^−1^ cm^−1^)	λ_em_/nm	*Φ*_oc_	*Φ*_co_	*Φ_f_*	*τ*_f_ (ns)	Solvent	*Ref.*
**50o**	289 (0.63), 342 (0.92), 417 (2.15)	502	0.39	0.0005	0.167	2.44	benzene	[50]
**51o**	287 (0.75), 364 (0.80), 434 (2.42)	517	0.35	0.0004	0.161	2.66	benzene	[50]
**52o**	298 (0.88), 450 (2.00)	513	-	-	0.02	< 0.5	benzene	[51]
**53o**	300 (0.90), 451 (2.30)	511	-	-	0.03	< 0.5	benzene	[51]
**54o**	316 (0.88), 460 (2.92), 490 (3.82)	513	-	-	0.06	< 0.5	benzene	[51]
**55o**	303 (0.68), 360 (0.99), 448 (2.90)	526	0.12	0.004	0.39	3.07	benzene	[51]
**56o**	305 (0.58), 372 (0.78), 460 (2.88)	529	0.12	0.002	0.40	2.94	benzene	[51]
**57o**	316 (0.65), 380 (0.64), 492 (4.42)	545	0.05	0.003	0.30	1.63	benzene	[51]
**58o**	300 (0.89), 383 (0.74), 466 (2.83)	545	0.15	0.004	0.32	3.19	benzene	[51]
**59o**	310 (0.99), 391 (0.93), 475 (4.23)	554	0.14	0.002	0.36	3.70	benzene	[51]
**60o**	318 (0.64), 405 (0.58), 510 (4.07)	557	0.03	0.004	0.34	2.04	benzene	[51]

λ_max_: absorption maximum; ε: molar absorption coefficient; λ_em_: fluorescence maximum; *Φ*_oc_: cyclization quantum yield; *Φ*_co_: cycloreversion quantum yield; *Φ*_f_: fluorescence quantum yield. *τ*_f_: fluorescence lifetime.

**Table 10:** Photophysical and photochemical parameters of **61o**–**68o** in benzene solution.

Compd.	λ_max_/nm (ε/10^4^ M^−1^ cm^−1^)	λ_em_/nm	*Φ*_oc_	*Φ*_co_	*Φ_f_*	*τ*_f_ (ns)	Solvent	*Ref.*
**61o**	295 (0.69), 402 (2.87)	496	-	-	0.007	2.09	benzene	[52]
**62o**	292(0.61), 399 (2.76)	478	0.022	0.012	0.247	1.00	benzene	[52]
**63o**	292 (0.86), 393 (3.30)	473	0.012	0.009	0.097	0.59	benzene	[52]
**64o**	292 (0.90), 397 (3.31)	468	0.027	0.016	0.262	1.03	benzene	[52]
**65o**	295 (0.52), 424 (2.18)	543	0.005	0.004	0.014	< 0.5	benzene	[52]
**66o**	291 (0.76), 417 (2.85)	522	0.025	0.005	0.178	1.52	benzene	[52]
**67o**	293 (0.75), 416 (2.47)	506	0.018	0.002	0.585	2.63	benzene	[52]
**68o**	295 (0.61), 420 (2.37)	505	0.038	0.014	0.556	2.27	benzene	[52]

λ_max_: absorption maximum; ε: molar absorption coefficient; λ_em_: fluorescence maximum; *Φ*_oc_: cyclization quantum yield; *Φ*_co_: cycloreversion quantum yield; *Φ*_f_: fluorescence quantum yield; *τ*_f_: fluorescence lifetime.

**Table 11:** Photophysical and photochemical parameters of **69o**–**76o** in toluene solution.

Compd.	λ_max_/nm (ε/10^4^ M^−1^ cm^−1^)	Conversion Yield (%)	*Φ*_oc_	*Φ*_co_	Solvent	*Ref.*
**69o**	-	-	0.17	0.05	toluene	[54]
**70o**	-	-	0.15	0.04	toluene	[54]
**71o**	328, 390	-	0.27	<1.0 × 10^−5^	toluene	[54]
**72o**	- (0.33 at 360 nm)	-	-	-	toluene	[55]
**73o**	- (1.17 at 360 nm)	-	-	-	toluene	[55]
**74o**	- (0.53 at 356 nm)	42.3	0.106	0.014	toluene	[56]
**75o**	- (1.36 at 356 nm)	24.3	0.106	0.002	toluene	[56]
**76o**	- (0.78 at363 nm)	74.8	0.784	0.034	toluene	[56]

λ_max_: absorption maximum; ε: molar absorption coefficient; conversion yield: conversion ratio from open form to closed from at PSS state; *Φ*_oc_: cyclization quantum yield; *Φ*_co_: cycloreversion quantum yield.

**Table 12:** Photophysical and photochemical properties of **81o**–**92o** in hexene solution.

Compd.	λ_max_/nm (ε/10^4^ M^−1^ cm^−1^)	λ_em_/nm (*I*_fl_, a.u.)	*Φ_f_*	Solvent	*Ref.*
**81o**	446 (0.70)	531 (680)	0.20	hexane	[59]
**82o**	445 (0.77)	534 (590)	-	hexane	[59]
**83o**	456 (0.70)	531 (630)	-	hexane	[59]
**84o**	452 (0.73)	535 (610)	-	hexane	[59]
**85o**	447 (0.91)	532 (640)	-	hexane	[59]
**86o**	446 (0.89)	533 (610)	-	hexane	[59]
**87o**	455 (0.88)	535 (710)	-	hexane	[59]
**88o**	452 (0.87)	528 (400)	-	hexane	[59]
**89o**	446 (0.81)	532 (670)	-	hexane	[59]
**90o**	443 (0.82)	535 (420)	-	hexane	[59]
**91o**	455 (0.80)	530 (610)	-	hexane	[59]
**92o**	450 (0.71)	546 (395)	-	hexane	[59]

λ_max_: absorption maximum; ε: molar absorption coefficient; λ_em_: fluorescence maximum; *Φ*_f_: fluorescence quantum yield.

**Table 13:** Photophysical and photochemical parameters of **94o**–**96o** in hexane solution.

Compd.	λ_max_/nm (ε/10^4^ M^−1^ cm^−1^)	λ_em_/nm	*Φ_f_*	*τ*_fl_/ns (CH_3_CN)	Solvent	*Ref.*
**94o**	400 (3.00)	450	0.003	2.8	hexane	[61]
**95o**	379 (2.90)	450	0.002	2.8	hexane	[61]
**96o**	406 (3.30)	450	0.003	2.8	hexane	[61]

λ_max_: absorption maximum; ε: molar absorption coefficient; λ_em_: fluorescence maximum; *Φ*_f_: fluorescence quantum yield; *τ*_f_: fluorescence lifetime.

**Table 14:** Photophysical and photochemical properties of **94o**–**96o** in MeCN solution.

Compd.	λ_max_/nm (ε/10^4^ M^−1^ cm^−1^)	λ_em_/nm	Stokes Shift, Δν_fl_ (cm^−1^)	Solvent	*Ref.*
**97o**	342 (1.64), 417 (1.36)	547	5700	MeCN	[62]
**98o**	364 (1.88), 414 (1.84)	544	5750	MeCN	[62]
**99o**	340 (2.00), 418 (1.72)	541	5450	MeCN	[62]
**100o**	332 (0.68), 414 (1.04)	533	5400	MeCN	[62]
**101o**	343 (1.44), 421 (1.24)	510	4100	MeCN	[62]
**102o**	275 (4.68), 460 (0.53)	560		MeCN	[63]
**103o**	263 (3.03), 270 (3.28), 450 (2.85)	560		MeCN	[63]

λ_max_: absorption maximum; ε: molar absorption coefficient; λ_em_: fluorescence maximum.

**Table 15:** Photophysical and photochemical parameters of **104o**–**105o** in MeCN solution.

Compd.	λ_irr_/nm	*Φ* [at λ_irr_] Degassed	*Φ* [at λ_irr_] Non-Degassed	PSS [at λ_irr_] (open:closed)	Solvent	*Ref.*
**104o**	313 (o→c) 405 (o→c) 577 (o→c)	0.18 0.30 0.0003	0.005 0.009 0.0003	34:66 0:100 100:0	MeCN	[74]
**105o**	289 (o→c) 365 (o→c) 405 (o→c) 577 (o→c)	0.49 0.55 0.55 0.063	0.54 0.51 0.41 0.06	0:100 0:100 0:100 100:0	MeCN	[75]

λ_irr_: light irradiation wavelength; *Φ*_oc_: cyclization quantum yield; PSS: conversion ratio from open form to closed from in the PSS state.

**Table 16:** Photophysical and photochemical parameters of **104o**–**105o** in solution.

Compd.	λ_max_/nm (ε/10^4^ M^−1^ cm^−1^)	*Φ_oc_*	*Φ_co_*	solvent	*Ref.*
**113o**	356 (1.5)	1.9 × 10^−4^	-	1.4-dioxane	[90]
**113c**	260 (2.9)	-	0.55	1.4-dioxane	[90]
**114o**	317 (0.5)	0.01	-	1.4-dioxane	[90]
**114c**	284 (0.6)	-	0.19	1.4-dioxane	[90]

λ_max_: absorption maximum; ε: molar absorption coefficient; *Φ*_oc_: cyclization quantum yield; *Φ*_co_: cycloreversion quantum yield.

## Data Availability

Not applicable.
